# New access to intraluminal gastric pathologies: the SP da Vinci transgastric approach

**DOI:** 10.1007/s00464-026-12829-y

**Published:** 2026-05-07

**Authors:** Franziska Renger, Alessandro Francesco Armienti, Yulia Brecht, Vladimir J. Lozanovski, Edin Hadzijusufovic, Hauke Lang, Peter Philipp Grimminger

**Affiliations:** 1 Department of General-, Visceral-, and Transplantation Surgery, University Medical Center of the Johannes Gutenberg-University Mainz, Langenbeckstraße 1, 55131 Mainz, Germany; 2 Department of Surgery, University Vita-Salute San Raffaele Milan, Milan, Italy

**Keywords:** Da Vinci robot-assisted gastric surgery, Minimally invasive surgery, Transgastric, Robotics, Single port, Organ preserving

## Abstract

**Background:**

Surgical management of gastric submucosal tumors in anatomically challenging sites such as the gastroesophageal junction (GEJ), fundus, or proximal lesser curvature remains technically demanding. The da Vinci single-port (SP) robotic platform may provide a minimally invasive, organ-preserving alternative by enabling stable endoluminal access and precise dissection.

**Methods:**

We conducted a case series of five patients who underwent SP robot-assisted transgastric resections for circumscribed gastric pathologies between May and August 2025. Patient demographics, tumor characteristics, operative details, pathology, and short-term outcomes were analyzed.

**Results:**

Two patients presented with gastric metastases from malignant melanoma, one with a neuroendocrine tumor (NET), one with a gastrointestinal stromal tumor (GIST), and one with a leiomyoma. All lesions were located in anatomically challenging areas and ranged in size from < 1 cm to 5.5 cm. Four patients underwent submucosal dissection, and one required full-thickness resection. Operative times ranged from 66 to 134 min. Median console time was 38 min. No bleeding, conversion, tumor rupture, or spillage occurred. Patients were discharged between postoperative day (POD)-3 and POD-7, with no reoperations, morbidity, or 30-day mortality. Histopathology confirmed R0 resection of one GIST and both melanoma metastases; the NET G2 had positive margins but very low proliferative activity (Ki-67 < 0.1%).

**Conclusions:**

Da Vinci SP robot-assisted transgastric resection is a feasible and safe organ-preserving technique for GEJ and gastric tumors. The platform offers great exposure, stable endoluminal access, and three arms for dissection. This technique complements established endoscopic and laparoscopic procedures. The presented method could also be combined with extraluminal lymphadenectomy. Larger studies with long-term follow-up will define optimal indications and the role of this innovative approach within minimally invasive gastric surgery.

**Supplementary Information:**

The online version contains supplementary material available at 10.1007/s00464-026-12829-y.

Endoscopic and surgical treatment of gastric lesions in anatomically challenging locations has ever been a challenge. Anatomically challenging locations in this regard are the proximity of the gastroesophageal junction, the fundus and the lesser curve [1]. These lesions encompass gastric or junctional submucosal tumors, predominantly GISTs, but also mucosal metastases. According to the European Society of Gastrointestinal Endoscopy guidelines, gastric lesions may be managed endoscopically or require surgical resection, depending on size, morphology, location, margin delineation and estimation of invasion depth [2]. Typically, extended lymphadenectomy or wide margins should not be required. Endoscopic treatment execution can be constrained by the lesions’ ‘inner gastric’ location, as some endoscopic methods require straight-line access with the endoscope [3]. In such scenarios, surgical resection becomes necessary.

To limit surgical trauma, various approaches such as endoscopic, laparoscopic, laparo-endoscopic, single-port/single-incision laparoscopic and robotic procedures have been developed [1, 4–9]. However, no standard approach has been established indicating that ‘the technique’ is still to be found.

With the introduction of the da Vinci SP robotic platform (Intuitive Surgical, Sunnyvale, CA, USA), new and improved technical options regarding angulation, visualization and maneuvering 3 arms in narrow spaces have emerged. Compared with conventional laparoscopic transgastric or laparo-endoscopic approaches, the SP platform offers stable endoluminal exposure, true three-arm triangulation within the gastric lumen, frontal visualization of target lesions, and advanced suturing capabilities. Also, the need for additional intraoperative endoscopy is reduced.

The safety and feasibility of SP procedures have been demonstrated in urologic and gynecological surgery, [10–13] but also in abdominal colorectal surgery [14–16]. In upper gastrointestinal surgery, the use of SP has been initially explored in gastric procedures, with early experiences of distal and total gastrectomy for malignant disease [17, 18].

The use of natural cavities as surgical fields may reduce surgical trauma and facilitate organ-preserving approaches. In the present technique, transgastric access is established via a mini laparotomy, enabling controlled endoluminal work.

Within confined surgical fields such as small anatomical cavities, SP robotic platforms can provide the stability and precision required to achieve accurate dissection and oncological radicality. For example, the introduction of SP technology in transanal minimally invasive surgery was considered safe and promising, particularly for its ability to facilitate precise work within restricted pelvic spaces [19–21].

In upper gastrointestinal procedures, safety and feasibility of SP robot-assisted procedures have also been demonstrated, e.g., for distal gastrectomy [22] or esophagectomy [23].

We started using the da Vinci SP robotic system at our institution in 2024. Until now, we have performed far more than 100 cases for esophagogastric pathologies [23–26]. In 2025, we developed the SP transgastric surgery approach for the treatment of intraluminal gastric pathologies and present the technique and case series here.

## Materials and methods

### Patients and methods

All SP transgastric procedures were performed at the Department of General, Visceral, and Transplantation Surgery, University Hospital Mainz, using the da Vinci SP System (Intuitive Surgical Inc., Sunnyvale, CA, USA) and the corresponding instruments. All SP transgastric procedures were performed by one surgeon (PPG), who was very experienced in robotic upper GI surgery, and his team. Data were extracted from the written or electronic medical record. This retrospective case series was conducted in accordance with the principles of the Declaration of Helsinki. According to institutional regulations, formal IRB approval was not required. All patients provided written informed consent for the procedure and for the use of their clinical data for scientific publication.

All five patients underwent SP da Vinci robot-assisted transgastric resections for circumscribed gastric endoluminal pathologies between May 1st, 2025 and August 31st, 2025. All cases were initially planned for submucosal dissection based on preoperative assessment, including endoscopy and imaging, with particular consideration of the suspected layer of origin and growth pattern. (See Table 2 Detailed Case Descriptions) Submucosal dissection was reserved for size-limited lesions without clear evidence of muscularis propria invasion. Intraoperative conversion to full-thickness resection was performed if deeper wall involvement was suspected. The overall surgical aim was to achieve an R0 resection. Standard oncological principles for submucosal tumors were strictly adhered to, including preservation of the pseudocapsule, avoidance of tumor rupture or spillage.

All patients underwent multidisciplinary tumor board consultation. Demographic data including age and sex were collected. Perioperative data such as ASA score, precise tumor location within the stomach, size of the lesion, preoperative histology, TNM staging corresponding to the entity and prior endoscopic or systemic treatment were extracted. Operative data included the overall operation time, blood loss and intraoperative complications. Additionally, length of hospital stay, definitive histopathologic results, 30-day morbidity and 30-day mortality were assessed.

### Surgical technique

The surgical technique of the transgastric approach is demonstrated in our video.

### Operation room setup and patient positioning

**Fig. 1:**
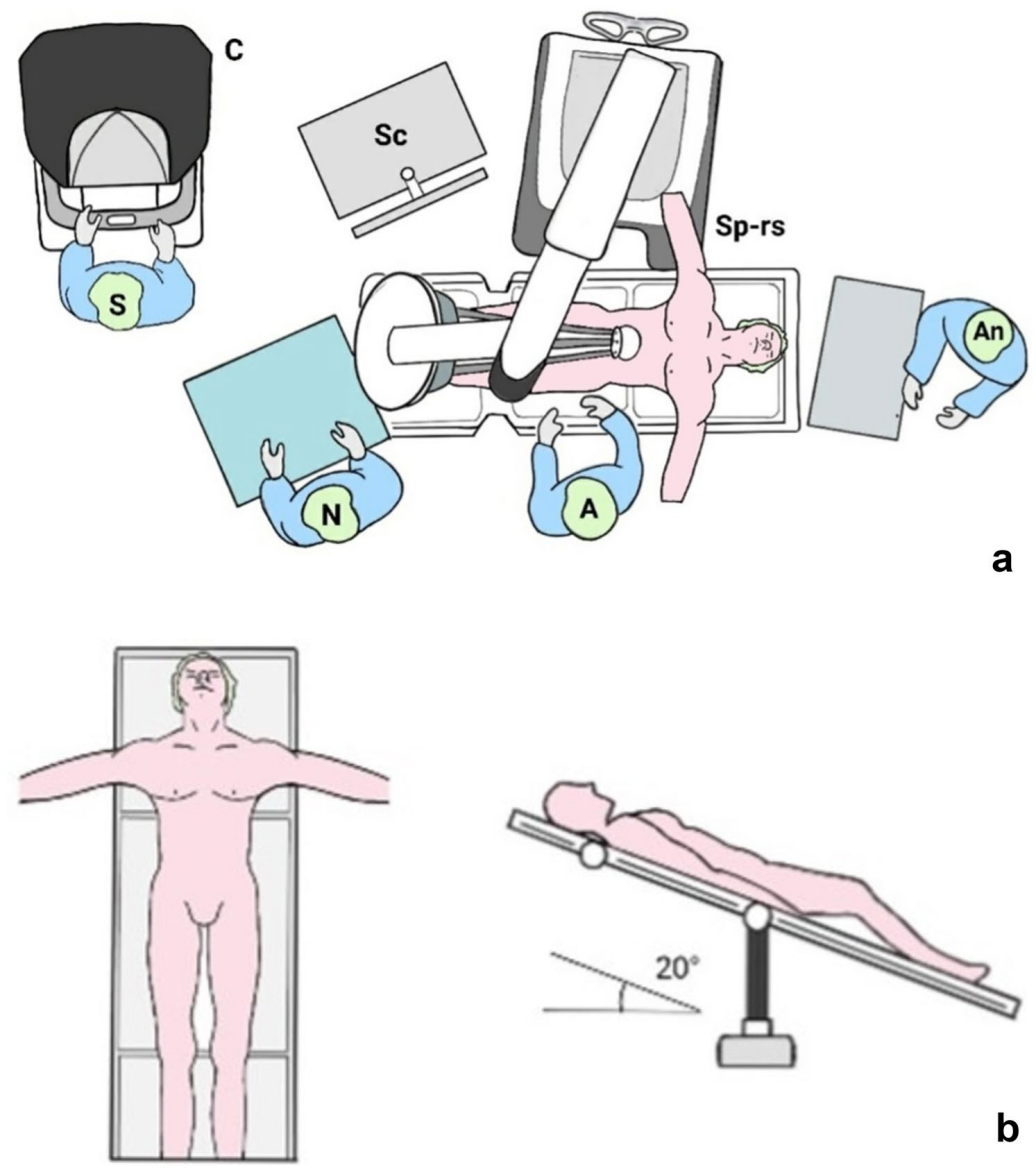
Setup: The da Vinci SP surgical cart and the vision cart are positioned on the patient’s right side, while the assistant surgeon is positioned on the patient’s left (**a**). The patient is placed in a supine and 20° anti-Trendelenburg position throughout the procedure (**b**)

The patient is placed in a supine, anti-Trendelenburg position. The da Vinci SP surgical cart and the vision cart are positioned on the patient’s right side, while the assistant surgeon is positioned on the patient’s left (Fig. 1a, b).

### Access to the abdominal cavity and the stomach

**Fig. 2:**
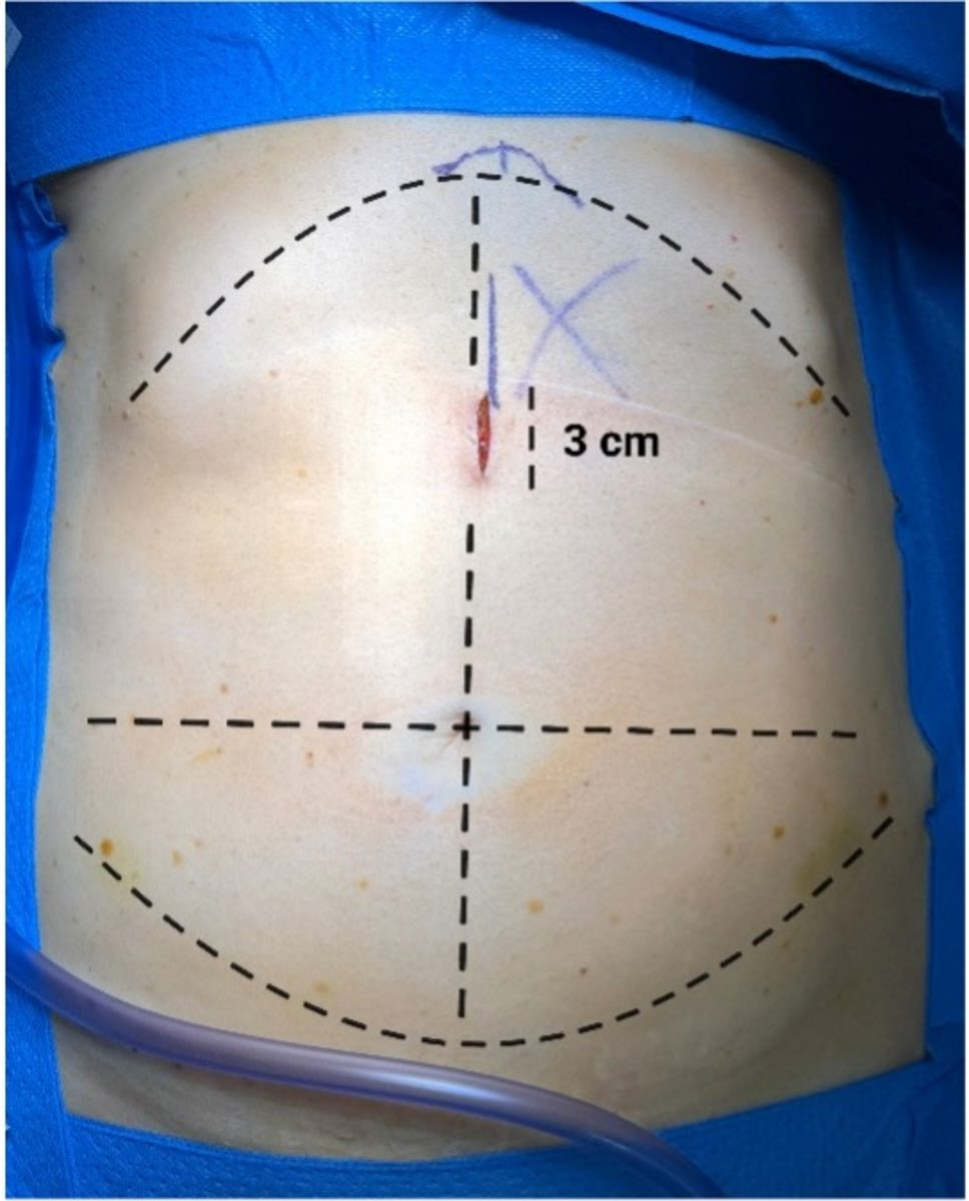
Access: A 3–4 cm supraumbilical midline incision is created. The precise location is determined according to the stomach’s position by reviewing the CT scan

A 3–4 cm supraumbilical midline incision is created. The precise location is determined according to the stomach’s position on preoperative CT imaging (Fig. 2).

### Access to the gastric cavity and docking

**Fig. 3:**
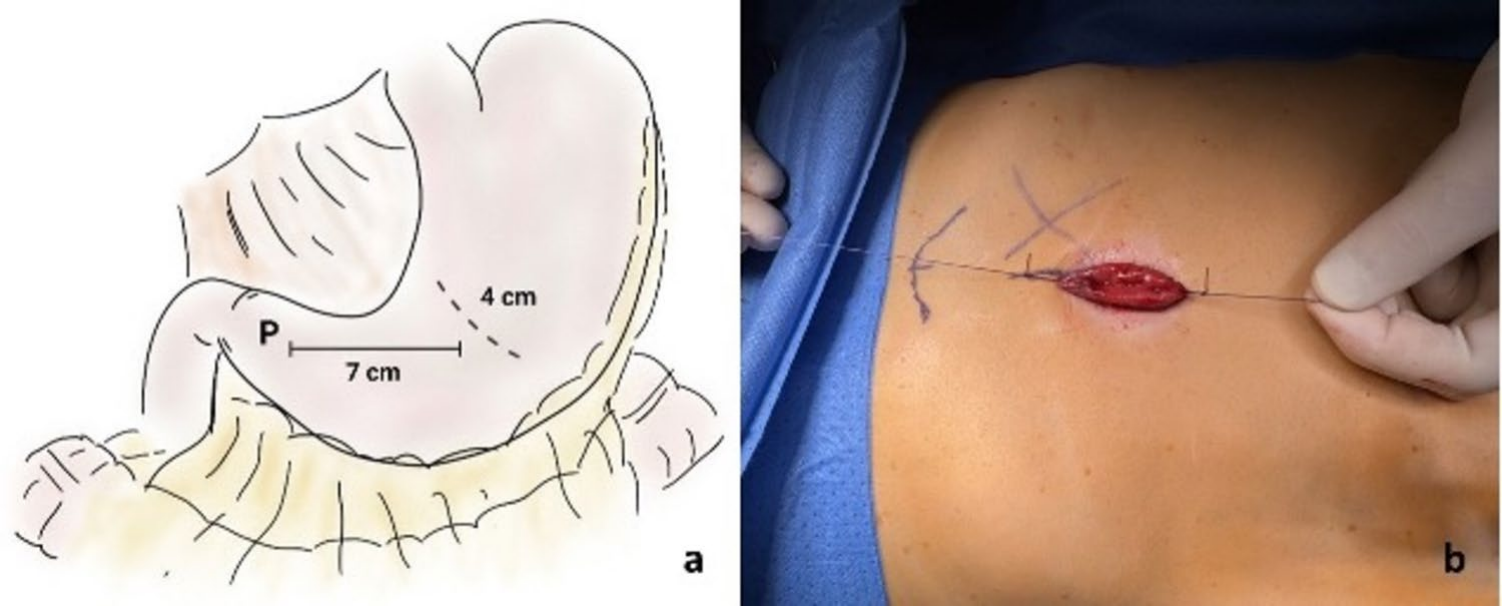
Access to the stomach**:** A gastrotomy is created at the transition between the gastric corpus and antrum, approximately 7 cm proximal to the pylorus (**a**). Two stay sutures secure the stomach to the abdominal wall (**b**). *P* pylorus

**Fig. 4:**
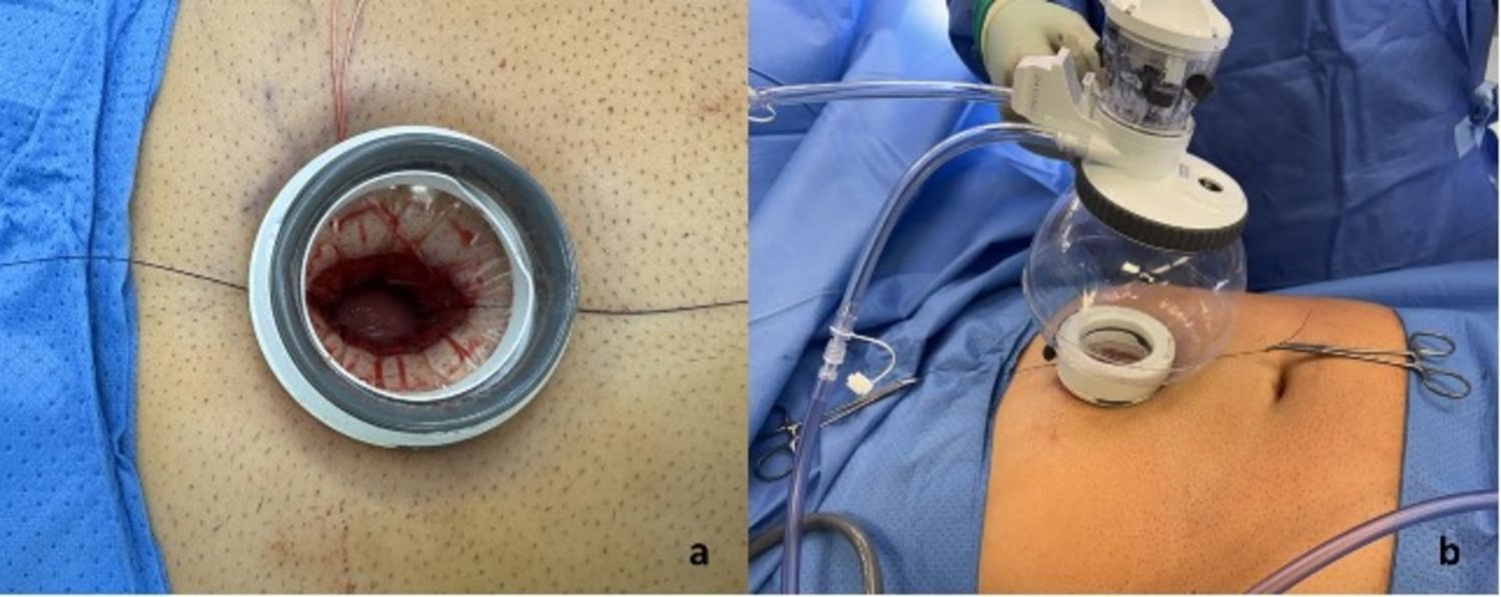
Docking: the wound protector is introduced directly into the gastric lumen (**a**), and insufflation establishes a stable endoluminal surgical field (**b**)

**Fig. 5:**
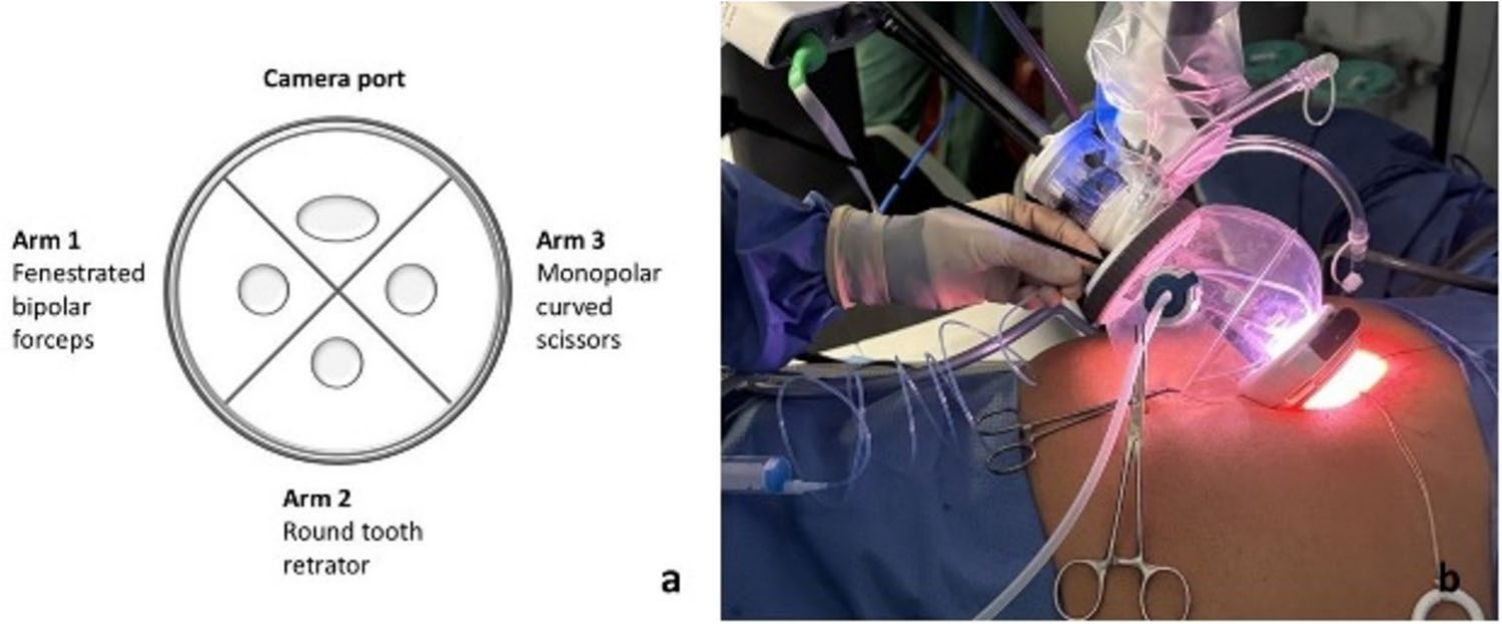
Instrument placement: Instruments may change port position to optimize traction and exposure, depending on the tumor’s location (**a**). Irrigation and suction can be achieved by introducing a tube through the accessory port (**b**)

A 4-cm transverse anterior gastrotomy is created approximately 7 cm proximal to the pylorus: The stomach is gently elevated and partially exteriorized through the incision, allowing direct palpation of the pylorus. Orientation is guided by the vessels along the greater and lesser curvature. The gastric wall is then lifted to the access site, and the gastrotomy is performed under direct visualization (Fig. 3a). Two stay sutures secure the gastrotomy to the abdominal wall and provide gastric traction (Fig. 3b). The gastrotomy is kept limited in size, allowing the gastric wall to be securely positioned within the wound retractor, whose radial tension stabilizes the gastric wall at the access site. The wound protector is introduced through the gastrotomy into the gastric lumen, after which the SP da Vinci access port and the short entry guide are positioned (Fig. 4a, b). To minimize contamination, gastric decompression is achieved with a nasogastric tube, and suction is used as needed during the procedure. Insufflation is initiated at 8–10 mmHg to establish a stable endoluminal surgical field. If necessary, a postpyloric loop can be placed during the setup phase to minimize intestinal inflation. The SP robotic system is subsequently docked. Instrument placement is as follows: fenestrated bipolar forceps are inserted through Arm 1, a round-tooth retractor through Arm 2, and monopolar curved scissors through Arm 3 (Fig. 5). The SP access provides an integrated accessory port which allows insertion of sutures, needles, gauzes, and other accessories while maintaining stable exposure.

### Exploration, identification of the target lesion and the cardia

**Fig. 6:**
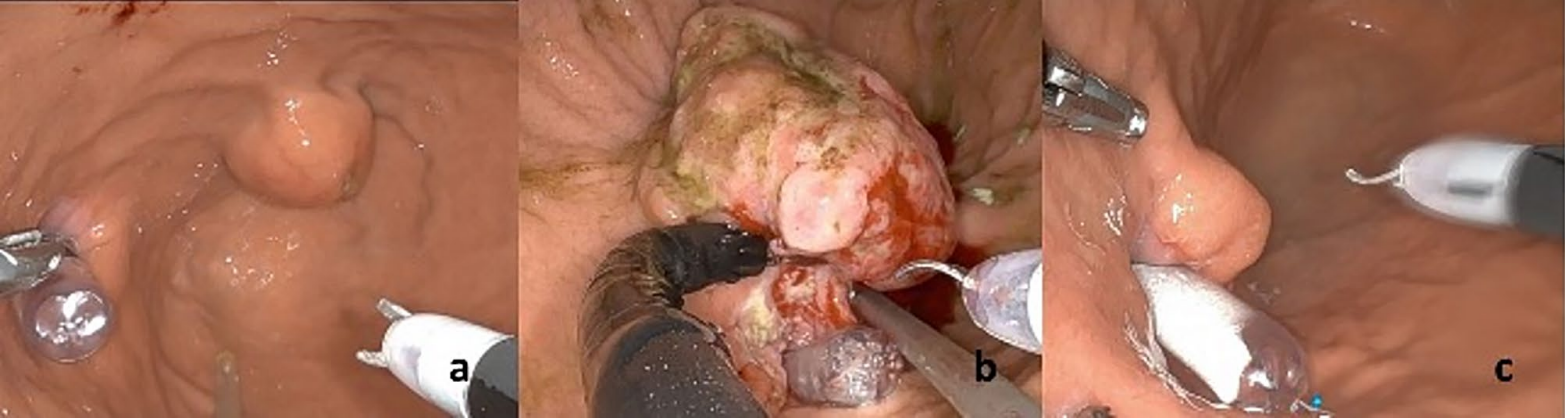
Intraoperative exposure of three different lesions and their relationship to the gastroesophageal junction, facilitated by gastric tube placement (cases 1–3, in order of appearance)

**Fig. 7:**
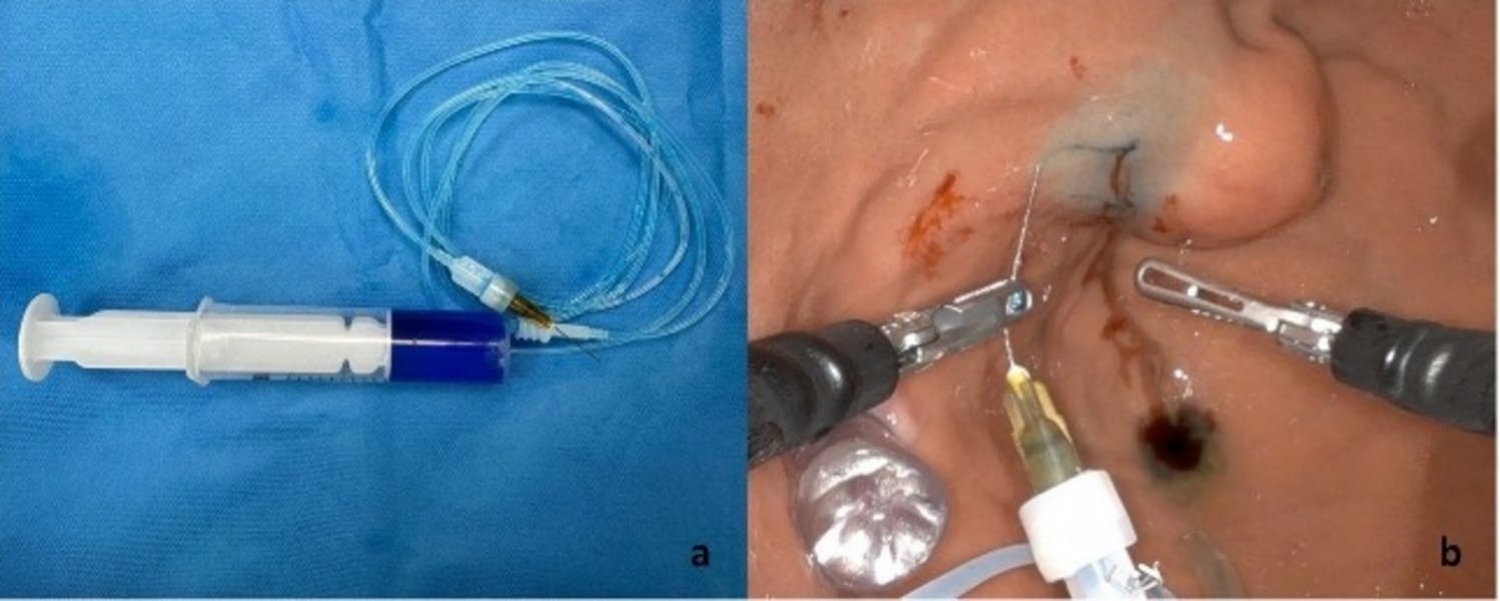
Submucosal lifting: a syringe with methylene blue is inserted through the accessory trocar of the insufflation cup (**a**), and a submucosal injection of methylene blue is performed to achieve lifting of the lesion (**b**)

A systematic inspection of the entire gastric mucosa is performed. The target lesion is identified, and an orogastric tube is introduced to localize the cardia and assess its relationship to the lesion. The SP transgastric setup provides direct, frontal visualization of anatomically challenging regions—including the area adjacent to the cardia, the fundus, and the lesser curvature—and facilitates optimal instrument alignment, enabling precise access to locations that are otherwise difficult to reach endoscopically (Fig. 6a–c). To achieve adequate lifting of the lesion, reduce the risk of perforation, and clearly delineate the interface between the submucosal and muscular layers, a submucosal injection is administered. A solution consisting of 1 mL of adrenaline (1 mg/dL) and 1 mL of methylene blue (10 mg/dL) is injected using an endoscopic syringe (Fig. 7a), resulting in satisfactory submucosal elevation (Fig. 7b).

### Submucosal dissection and evaluation of macroscopic radicality

**Fig. 8:**
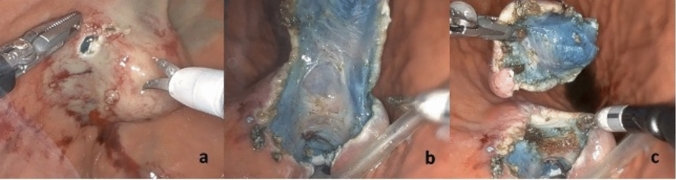
Submucosal dissection: the mucosa is incised at the base of the lesion (**a**), followed by stepwise separation of the submucosal from the muscular layer (**b**). Dissection is then continued circumferentially, ensuring an adequate safety margin (**c**)

Submucosal dissection of the lesion is performed with a bipolar device and monopolar scissors. Dissection is initiated at the mucosal incision site (Fig. 8a) and proceeds with stepwise separation of the submucosal layer, aided by applied traction to expose the underlying muscular layer (Fig. 8b). The resection bed, its completeness and integrity are verified (Fig. 8c).

### Mucosal closure

**Fig. 9:**
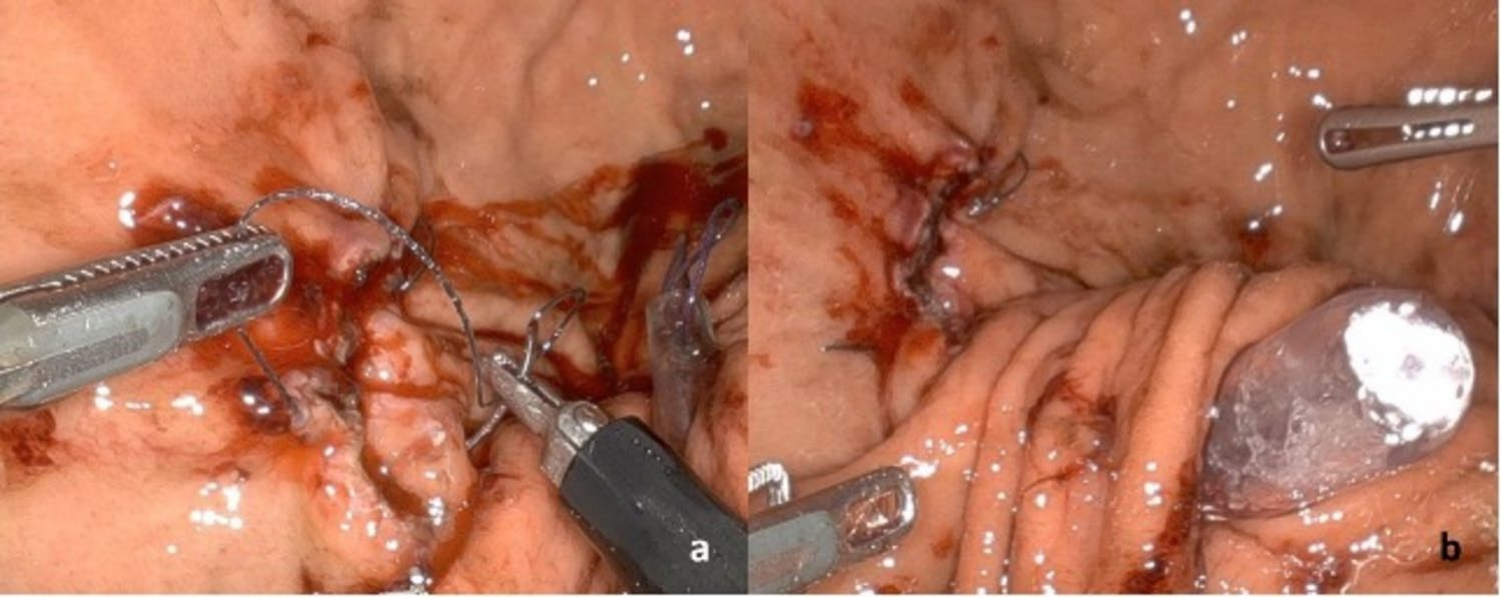
Suturing: an extended mucosal defect can be closed to prevent bleeding. The defect is closed with a continuous 4/0 V-Loc suture (**a**, **b**)

Absence of active bleeding and preservation of gastric wall integrity are confirmed. Mucosal and submucosal layers can be closed using a double continuous 4/0 V-Loc suture in a tension-free manner (Fig. 9a, b).

### Specimen retrieval

**Fig. 10:**
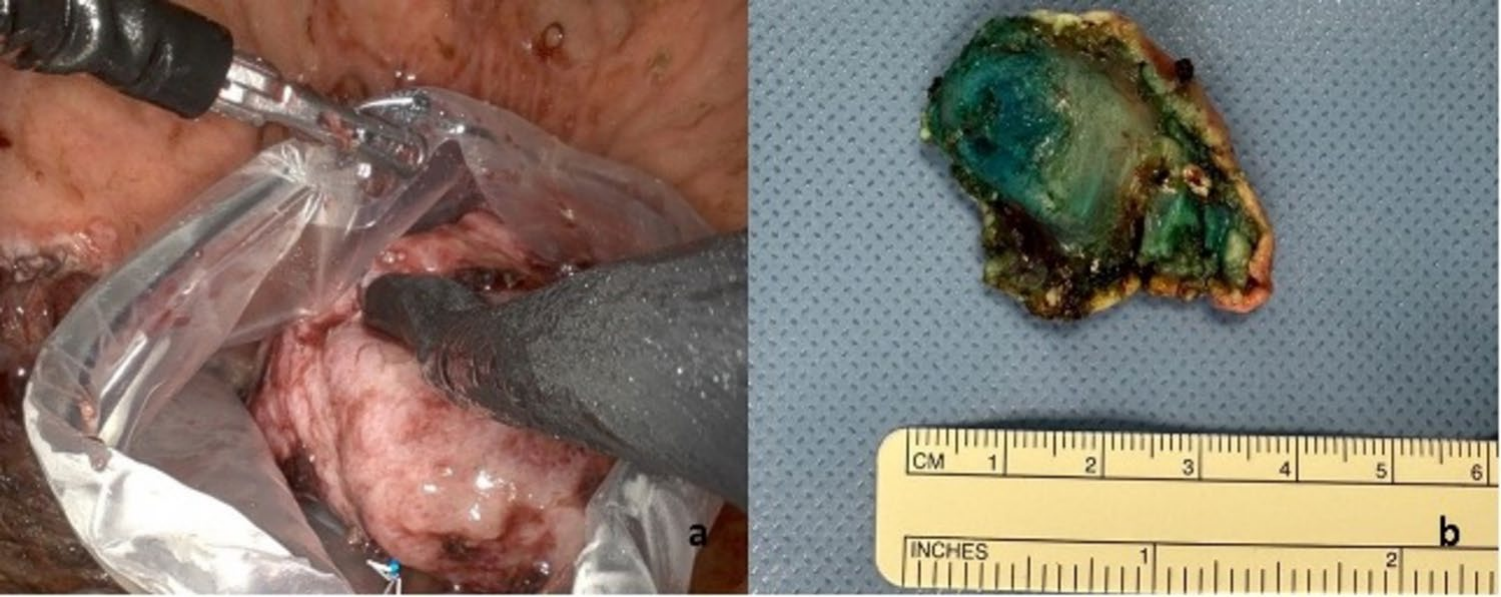
Specimen retrieval: the specimen is retrieved within an endobag to prevent tumor cell dissemination (**a**), and its integrity and radicality are verified macroscopically (**b**)

To prevent spillage and ensure safe specimen retrieval, the specimen is retrieved en bloc using an endobag through the access port (Fig. 10a), then macroscopically examined and measured extracorporeally (Fig. 10b).

### Closure

**Fig. 11:**
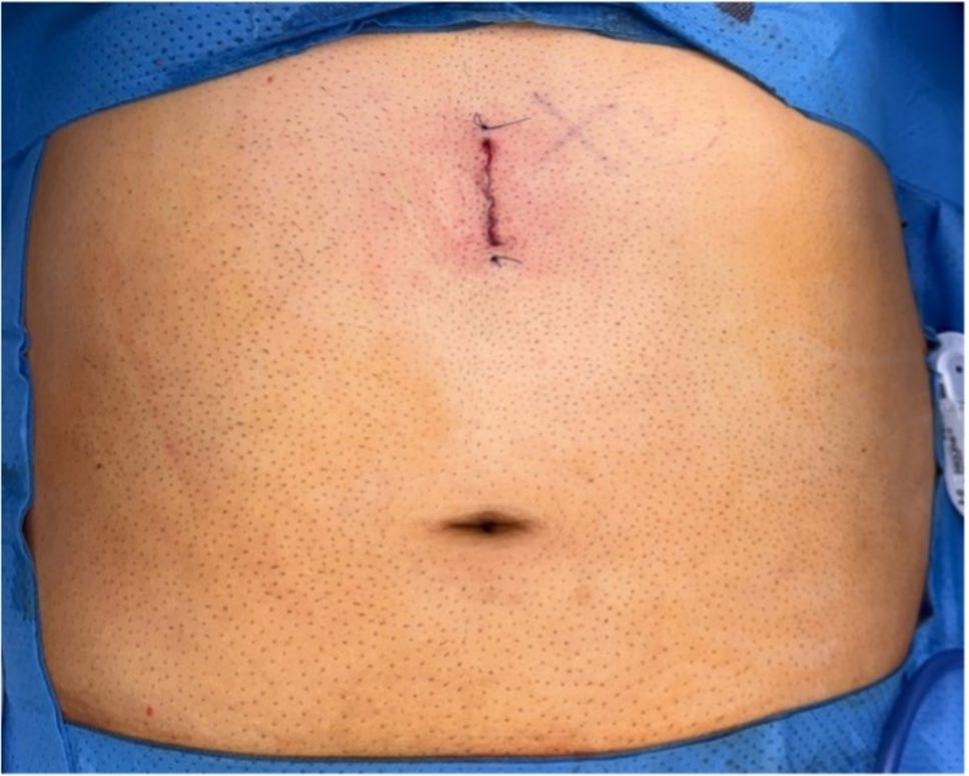
Skin closure

The robot system is dedocked and the stomach is detached from the abdominal wall. A nasogastric tube can be placed. The gastrotomy is closed using a single layer running 3/0 PDS under direct visualization, with stay sutures facilitating exposure. Routine leak testing is not performed. The mini laparotomy is closed in layers with 2/0 PDS, and the skin is approximated with an intradermic absorbable suture (Fig. 11).

### Postoperative assessment

Postoperative assessment was based on clinical evaluation, without routine contrast studies or endoscopy.

## Results

### Patient characteristics

**Fig. 12:**
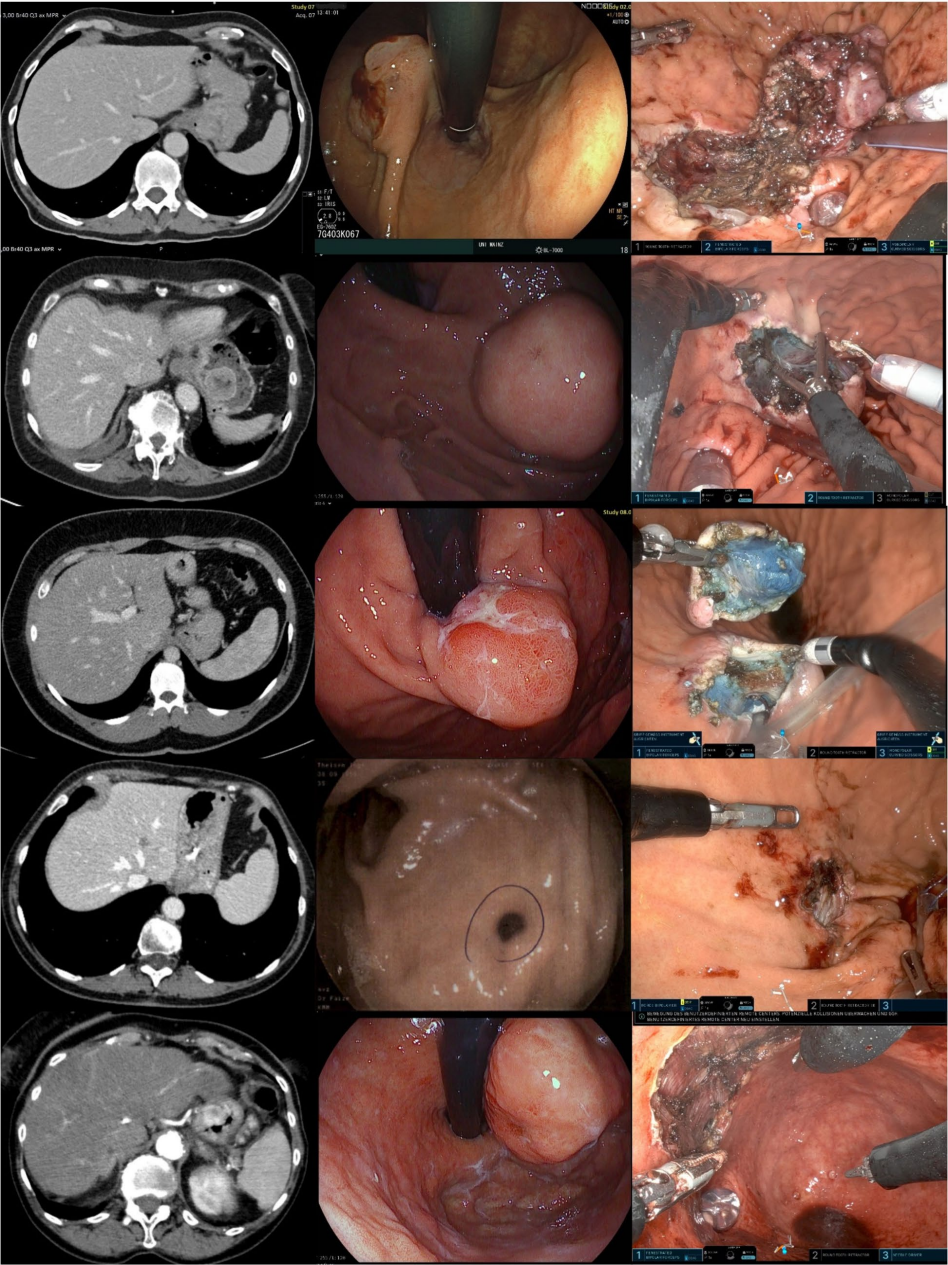
Representative preoperative imaging of the five cases, illustrating lesion location, presented in order of appearance

Patients’ characteristics are shown in Table 1. Detailed case descriptions are shown in Table 2. Corresponding imaging is shown in Fig. 12. Two patients were male, and ages varied from 45 to 83 years. Three patients were ASA III. Median BMI was 21. Tumors were located at anatomically challenging sites, predominantly within 2 cm of the GEJ, varying from less than 1 cm up to 5.5 cm in diameter. In 4 of our 5 cases, preoperative biopsies were confirmed in the definitive histopathologic workup. Two patients had gastric metastases from malignant melanoma, one had a NET G2 within 2 cm of the GEJ, one had a GIST, and one had a suspected GIST that was ultimately diagnosed as a leiomyoma. Three patients had significant comorbidities. Two patients had metastatic melanoma disease. No patient underwent primary endoscopic resection. All cases were reviewed in a multidisciplinary tumor board, which recommended surgical resection.

**Table 1:** Patients’ characteristics

	Patient				Diagnostic workup					Systemic therapy		Surgery			LOS	Histology	30-days morbidity	30-days mortality
	Age	Sex	ASA	BMI	Tumor location	Diameter (cm)	Biopsy	Staging	ER		OT (min)	CT (min)	BL (ml)	IC				
Case 1	60	M	III	21	GEJ within 2 cm	5.5 × 5	melanoma metastasis	cTxN + M +	No	Dabrafenib, Trametinib, Nivolumab,	134	106	0	No	7	melanoma metastasis, R0	No	No
Case 2	83	F	III	24	GEJ within < 2 cm	3 × 3	GIST	cT2N0M0	No	no	79	39	0	No	4	GIST no mitosis/HPF, R0	No	No
Case 3	45	M	I	32	GEJ within < 2 cm	2.5 × 2.5	negative for malignancy	cTxN0M0	No	no	66	28	0	No	3	leiomyoma	No	No
Case 4	66	F	II	20	fundus	< 1	melanoma metastasis	TxN0M +	No	no	68	27	0	No	3	negative for malignancy	No	No
Case 5	74	F	III	18	corpus	3.4 × 2.5	NET	cTxN0M0	No	no	91	38	0	No	4	NET, G2, R1, Ki 67 < 1%	No	No

*ER* endoscopic resection *OT* operation time *CT* console time, *BL* blood loss *IC* intraoperative complication *LOS* length of stay (postoperative)

**Table 2:** Detailed case descriptions

Case 1: A 60-year-old male with a history of acral lentiginous melanoma diagnosed in 2005 underwent multimodal treatment including interferon-alpha therapy, lymph node dissection, and radiotherapy. Metachronous pulmonary metastases were treated with BRAF inhibitors for nearly 10 years. In 2024, liver metastases prompted a switch to combination immunotherapy, complicated by immune-related colitis. In 2025, the patient developed progressive dysphagia and acute abdominal pain. Endoscopy revealed a bleeding 2 × 2 cm exophytic mass in the gastric cardia; biopsies were in keeping with a melanoma metastasis. Endoscopic ultrasound (EUS) showed submucosal, but no muscular involvement. Re-Staging CT scan revealed a growing gastric mass of 5 × 5 cm diameter while liver metastases remained stable. Multidisciplinary tumor board (MTB) advised for a limited surgical resection, considering the patient’s weakened general condition Thus, a transgastric robotic resection—an endoluminal submucosal dissection—was performed. Postoperatively, no complications occurred, and the patient was discharged on postoperative day 7 due adjustment of chronic pain therapy by the pain management team. Histology confirmed metastatic melanoma with clear margins (< 0.1 cm)
Case 2: In an 83-year-old female with a complex medical history, including atrial fibrillation, arterial hypertension, nodular goiter, mild aortic sclerosis, and severe microangiopathic leukoencephalopathy, an incidental gastric lesion suspicious for a GIST was identified on CT scan. Endoscopy confirmed a submucosal mass in the subcardial region along the lesser curve, measuring approximately 50 mm. The lesion had a central erosion, soft consistency, and easily liftable overlying mucosa, with no signs of active bleeding. Due to anticoagulant therapy, biopsies were deferred. A contrast-enhanced CT showed the lesion measuring 3.3 × 3.1 cm, without suspicious lymphadenopathy Multidisciplinary tumor board discussion advised surgical resection. An SP transgastric submucosal dissection was planned and intraoperatively converted to a wedge resection due to suspected deeper wall involvement. The patient was discharged on POD 4. Histopathological analysis confirmed a GIST measuring 3.5 × 2.9 × 2.0 cm. Resection margins were clear (R0), with a minimum margin of 0.1 cm. Mitoses were 0/HPF, low risk for progression
Case 3: A 45-year-old male, presented with dyspeptic symptoms. Endoscopy revealed a 1.5 × 2 cm junctional lesion; biopsies were inconclusive but negative for malignancy. EUS showed submucosal growth, and contrast-enhanced CT was unremarkable. A watch-and-wait approach was adopted. At follow-up in April 2025, the lesion had increased to 2.5 × 2 cm at the cardia (Z-line at 38 cm), with central ulceration suggestive of a GIST. Repeat biopsies remained negative. CT scan showed localized thickening of the gastric wall. Due to growth, ulceration, and ongoing symptoms, malignancy could not be ruled out. After multidisciplinary discussion, endoscopic removal was excluded because of the central ulceration, and a surgical resection was advised. An SP robot-assisted transgastric submucosal dissection both on the level of the esophagus and the stomach has been performed. The patient was discharged on postoperative day 4. Histologic workup showed a leiomyoma
Case 4: This case involves a 66-year-old female with a history of insulin-dependent diabetes mellitus, arterial hypertension, gastroesophageal reflux disease, and prior fundoplication via laparotomy. In 2012, she was diagnosed with malignant melanoma of the left eye. Due to local recurrence, complete exenteration of the left orbit was performed in 2019, followed by curative-intent radiotherapy. During a routine GERD follow-up in May 2025, endoscopy revealed a hyper pigmented lesion in the gastric fundus. Biopsies confirmed focal melanoma infiltration, and a clip was placed at the site for hemostasis. Staging with contrast-enhanced CT showed no additional metastatic disease. The case was reviewed by a multidisciplinary team, which recommended surgical resection, as endoscopic removal was deemed unsuitable. Surgical excision of the gastric lesion was successfully performed in 68 min without intraoperative complications. The patient had an uneventful postoperative course and was discharged on postoperative day 3. Final histopathological analysis revealed no residual malignant cells within nodular tissue, indicating a complete resection of the previously biopsied melanoma focus
Case 5: A 74-year-old female with significant comorbidities, including atrial fibrillation on oral anticoagulation, arterial hypertension, chronic heart failure, aortic valve replacement, and an episode of acute renal failure underwent endoscopy in October 2024 for anemia workup. A lesion was identified in the subcardial region. Histological examination revealed a well-differentiated NET G2. Due to her general condition, major surgery was considered challenging. Additionally, she suffered from a traumatic shoulder fracture. After reconvalescence, a contrast-enhanced CT 3 months later confirmed the gastric NET—a contrast-enhancing mass below the cardia measuring 2.3 × 2.2 cm, stable in size. DOTATATE PET scan was also negative for lymph nodes and distant disease. Multidisciplinary oncological discussion opted intentionally for a limited minimally invasive approach without lymphadenectomy according to her general condition to control bleeding Preoperative endoscopy showed a subepithelial mass in the proximal gastric body close to the GEJ (41–44 cm from the dental arcade; GEJ at 39 cm), measuring approximately 3.0 × 2.5 cm. The patient underwent robotic SP transgastric submucosal resection, completed in 91 min without intraoperative or postoperative complications. She was discharged on postoperative day 4. Histopathological analysis confirmed a well-differentiated NET G2. The tumor measured 3.4 cm and involved the resection margin. Ki-67 was < 1%. Postoperatively, the case was discussed in a multidisciplinary tumor board specialized in neuroendocrine tumors. Given the low proliferative activity, a surveillance strategy with clinical and endoscopic follow-up after 3 months and CT imaging (thorax and abdomen) after 6 months was recommended

### Operative outcomes

Operative time ranged from 66 to 134 min. The median console time was 38 min. Four patients underwent SP robot-assisted transgastric submucosal dissection, and one patient required full-thickness resection, intraoperatively. No relevant intraoperative blood loss occurred. Nasogastric tube placement was used to guide orientation and protect the GEJ. Methylene blue was injected into the submucosal layer to facilitate dissection, optionally. Dissection was executed using fenestrated bipolar forceps, a round-tooth retractor and monopolar curved scissors. All procedures were carried out as organ-preserving procedures, as planned. No conversion to laparoscopy, tumor rupture, or spillage occurred.

### Pathology

Histopathological analysis confirmed the suspected pathology in four cases; one lesion was diagnosed as leiomyoma. One GIST was resected R0 with low risk of progression. Both melanoma metastases were resected R0. The NET G2 was resected with positive margins but demonstrated a very low proliferation index (Ki-67 < 0.1%).

### Postoperative course

Patients were discharged between POD-3 and POD-7. One patient required adjustment of chronic pain medication during hospitalization. No reoperations, surgical complications, or 30-day mortality occurred.

## Discussion

In this case series, we demonstrate that SP robot-assisted transgastric resection is technically feasible for selected intraluminal gastric lesions in anatomically challenging locations.

Our findings provide a basis for comparison with previous reports. Pathologies in anatomically challenging locations have been managed using various strategies, including laparoendoscopic, single-incision laparoscopic intragastric, laparoscopic transgastric, and da Vinci Xi robot-assisted transgastric approaches [1, 4–8]. Common oncological prerequisites are limited resections, small- to medium-sized tumors, no indication for lymphadenectomy, achievement of clear margins, avoidance of tumor spillage, and safe specimen retrieval.

The conventional laparoscopic approach is considered unsuitable for proximal tumors, as it may require mobilization of the gastroesophageal junction, potentially compromising blood supply, vagal innervation, or gastric integrity [1].

In laparoendoscopic approaches, target lesions are identified by intraoperative endoscopy, followed by surgical exposure for optimal port placement. Multiple transgastric ports are often required [4, 8]. In an Australian series, most tumors were resected using laparoscopic staplers and were retrieved transorally or via an additional gastrotomy [5]. In Japan, single-incision intragastric surgery has been performed, stapler-transecting tumors using loops for countertraction [9]. An alternative technique everts the tumor-bearing gastric wall through the gastrotomy for resection [27].

Stiekema et al. introduced a laparoscopic transgastric strategy that involves a mini laparotomy, gastrotomy, insertion of a wound protector for transgastric access, and the additional use of a surgical glove to allow laparoscopic instrument placement [6]. This approach resembles our SP strategy; however, it has inherent limitations in laparoscopic angulation.

Foroutani described resection of a large GEJ leiomyoma using multiport da Vinci Xi transgastric endoluminal trocar placement, with the GEJ defect closure performed over a gastroscope to preserve lumen caliber and prevent stricture formation [7]. Guerra et al. presented a series of 46 GIST resections with the Xi robot after endoscopic ultrasound identification of the lesion. This approach resembles laparoendoscopy but provides superior angulation and suturing options comparable to robo-endoscopic techniques, although it does not represent a truly endoluminal strategy [28].

The da Vinci SP robotic platform by Intuitive Surgical Inc., Sunnyvale, CA, USA, builds upon these concepts and has been developed to further address limitations in confined anatomical spaces. The platform uses a 25-mm access cannula through which a 3D camera and three articulated working instruments are deployed. Both the robotic boom and trocar can rotate 360°, enabling access to all quadrants without patient or robot repositioning. Synchronized rotation and movement of the camera and working arms as a single unit facilitate surgical exposure. In contrast to conventional laparoscopic or laparo-endoscopic approaches, the SP system enables stable endoluminal access with three articulated instruments and direct frontal visualization, facilitating precise dissection and intracorporeal suturing within the gastric lumen.

In our institution, we regularly use the SP robotic technique in upper GI surgery, predominantly in SP robot-assisted cervical esophagectomy (SP RACE) and SP robot-assisted subcostal esophagectomy (SP SC RAMIE) procedures. We recently published the first results of our SP SC RAMIE experience and could demonstrate feasibility, safety and technical adequacy [23].

In this case series, access to the stomach via mini laparotomy, gastrotomy, and placement of a wound protection ring was achieved. Lesions were readily identified using the SP robotic camera, and exposure was facilitated by endoluminal CO₂ insufflation. A gastric tube provided additional guidance for orientation regarding the esophageal lumen. The operation field was consistently well visualized. Methylene blue was injected submucosally to facilitate dissection. The snake-like movements and enhanced angulation of the robotic arms allowed precise maneuvering and dissection, while en bloc rotation of the platform further improved exposure. Instrument interference was minimal. Specimen retrieval was performed intraluminally via the gastrotomy and mini laparotomy, protected by the access port.

Size-limited (sub)mucosal tumors not requiring lymphadenectomy or wide margins were the indications for transgastric resections in our series, including GIST, leiomyomas, and NET. Our series also included symptomatic isolated mucosal metastases of malignant melanoma. Apart from the lesion itself, patients’ comorbidities further influenced the indication, with the aim of achieving tumor control while minimizing surgical trauma and morbidity. All tumors were located in anatomically challenging areas. Tumor sizes ranged from < 1 cm to 5.5 cm, comparable with previously reported series of transgastric resections [1, 6, 28]. Most prior reports did not emphasize comorbidities or ASA status, likely reflecting a more oncological than technical focus. In Guerra’s series of 46 patients, only 17% were ASA III–IV [28].

Operative times ranged from 66 to 134 min. This is shorter than other series [1, 4, 6, 27, 28]. Only Stiekema’s single-port laparoscopic transgastric series (42–93 min) reported slightly shorter times [6]. Additional procedural steps in multiport approaches, such as transgastric trocar placement and intraoperative endoscopy or ultrasound, likely contribute to longer durations. Median console time was 38 min.

All resections were organ sparing, with no intraoperative bleeding, conversions, tumor rupture, or spillage. No reoperations, in-hospital mortality, or 30-day mortality occurred, and no patient required adjuvant treatment. Histopathology confirmed the preoperative diagnosis in four cases, while one lesion proved to be a leiomyoma. One GIST was resected R0 with low risk of progression, both melanoma metastases were resected R0, and the NET G2 was resected with positive margins but had an extremely low proliferation index (Ki-67 < 0.1%).

Postoperatively, length of stay ranged from 3 to 7 days, consistent with previous series. The patient discharged on POD-7 required adjustment of chronic pain medication. However, length of stay as a parameter is traditionally influenced by national reimbursement incentives and outpatient infrastructure.

As with precursor techniques, our results suggest that SP robot-assisted transgastric resections are surgically feasible and may represent a promising organ-preserving approach in carefully selected patients.

To the best of our knowledge, this study presents the first surgical and simultaneous endoluminal submucosal dissection approach to tumors located at anatomically challenging gastric sites. The technique is organ-preserving and avoids gastroesophageal junction mobilization or devascularization, as the resection is performed from within the lumen. Thus, gastric motility and function are likely to remain unaffected.

The SP robotic setup allowed direct intraluminal visualization, eliminating the need for additional intraoperative endoscopy. All procedures were performed safely, with no intra- or postoperative complications reported. Notably, in Case 5, the patient was deemed unfit for major gastric resection by the multidisciplinary board, yet tumor and symptom control (bleeding) could still be achieved through this minimally invasive approach.

Our series demonstrated reasonable operative times. All procedures have been performed by an already experienced team in robotic SP procedures [23–25].

Several limitations are to be considered. Our case series included a heterogeneous group of tumor entities. The indication for a limited resection of a NET G2 was a trade-off of operability and the standard approach of resection with lymphadenectomy. A positive margin with a very low proliferation rate was accepted due to significant comorbidities. As such, this reflects an individualized decision rather than a generalized oncological strategy.

The small sample size and selected nature of our series limit generalizability. Furthermore, all procedures were performed in 2025 by a single surgeon with extensive laparoscopic and robotic upper GI experience [24, 25]. A higher BMI may increase technical difficulty; this should be considered during patient selection.

Follow-up data on long-term oncological or functional outcomes are not yet available. Thus, our conclusions remain preliminary and hypothesis-generating.

Oncologic conclusions are limited by the small and heterogeneous cohort, as well as the presence of a positive resection margin in one NET G2 case. Therefore, this approach should currently be considered as exploratory with respect to oncological outcomes.

Finally, the SP robotic platform itself is still in early clinical development. Accordingly, our report represents a snapshot of the technique’s early application and ongoing evolution.

As an outlook, we see considerable potential for SP robot-assisted transgastric surgery. This organ-preserving and function-preserving approach may spare patients from proximal gastrectomies, thereby reducing procedure-related morbidity, such as postoperative weight loss and reflux.

The SP system offers a stable operative field, three articulated working arms, high-definition vision, and suturing options beyond what is possible with endoscopic techniques alone, while still maintaining the possibility of conversion to standard laparoscopic surgery if necessary. Applications could range from endoscopic-like procedures (EMR, ESD) to wedge resections, expanding the therapeutic spectrum.

Importantly, indications initially should remain restricted to size-limited, submucosal tumors in locations where wide margins or lymphadenectomy are not required. This highlights the importance of multidisciplinary collaboration with endoscopists and gastroenterologists to determine the most appropriate access route for each patient.

Finally, as cancer care evolves with immunotherapy and checkpoint inhibitors, treatment resembles chronic disease treatment. In this context, the balance between oncological radicality and surgical morbidity becomes increasingly relevant. For frail, multi-therapy patients, the SP robot-assisted transgastric approach may offer a tailored compromise between disease and/or symptom control and minimal surgical trauma.

Future studies with larger cohorts and long-term follow-up are required to better define indications and oncological outcomes. The role of this innovative technique within minimally invasive gastric surgery remains to be established.

## Supplementary Information

Below is the link to the electronic supplementary material.

Supplementary file1 (MOV 77601 KB)

## Abbreviations

ASA: American Society of Anesthesiologists
BRAF: BRAF proto-oncogene
CT: Computed tomography
EMR: Endoscopic mucosal resection
ESD: Endoscopic submucosal dissection
EUS: Endoscopic ultrasound
GERD: Gastroesophageal reflux disease
GEJ: Gastroesophageal junction
GIST: Gastrointestinal stromal tumor
HPF: High-power field
MTB: Multidisciplinary tumor board
NET: Neuroendocrine tumor
PDS: Polydioxanone
POD: Postoperative day
SP: Single port
SP RACE: Single port robot-assisted cervical esophagectomy
SP SC RAMIE: Single port robot-assisted subcostal esophagectomy
TNM: Tumor, node, metastasis
